# Optical Coherence Tomography Biomarkers in Rhegmatogenous Retinal Detachment: A Narrative Review

**DOI:** 10.3390/jcm15135265

**Published:** 2026-07-06

**Authors:** Vladimir Milutinovic, Borivoje Savic, Jelena Kostic, Dragan Spaic, Bozidar Savic

**Affiliations:** 1 Clinic for Eye Diseases, University Clinical Center of Serbia, 11000 Belgrade, Serbia; 2 Department of Primary Health Care and Public Health, Faculty of Medicine, University of East Sarajevo, 73300 Foca, Bosnia and Herzegovina; 3 Institute of Veterinary Medicine of Serbia, Janisa Janulisa 14, 11000 Belgrade, Serbia

**Keywords:** rhegmatogenous retinal detachment, optical coherence tomography, OCT biomarkers, ellipsoid zone, external limiting membrane, visual prognosis, retinal imaging, epiretinal membrane

## Abstract

Rhegmatogenous retinal detachment (RRD) is a vision-threatening retinal disorder in which successful anatomical reattachment does not always result in satisfactory functional visual recovery. Optical coherence tomography (OCT) has become an essential non-invasive imaging modality for evaluating retinal microstructure and identifying biomarkers associated with postoperative visual outcomes. This narrative review summarizes and critically evaluates current evidence regarding the prognostic significance of OCT biomarkers following RRD repair. Available studies indicate that primary outer retinal biomarkers, particularly the integrity of the ellipsoid zone (EZ) and external limiting membrane (ELM), are the most reliable predictors of postoperative best-corrected visual acuity. Preservation of these structures reflects photoreceptor viability and is strongly associated with improved visual recovery. Secondary biomarkers, including hyperreflective foci, intraretinal cystic changes, retinal folds, persistent subretinal fluid, and preretinal hyperreflective dots, provide complementary information regarding inflammatory activity, retinal remodeling, and postoperative complications such as epiretinal membrane formation. Although these biomarkers have considerable clinical value for prognostication, patient counseling, and postoperative monitoring, their interpretation is limited by heterogeneity among studies, differences in imaging protocols, and the lack of standardized assessment criteria. Future large-scale prospective studies are needed to validate OCT biomarkers and establish standardized approaches for their integration into routine clinical practice.

## 1. Introduction

Rhegmatogenous retinal detachment (RRD) is a vision-threatening retinal disorder and an important cause of potentially reversible visual impairment worldwide [1,2,3,4]. The condition occurs when a full-thickness retinal break allows liquefied vitreous to enter the subretinal space, resulting in separation of the neurosensory retina from the underlying retinal pigment epithelium (RPE) [1]. If left untreated, progressive photoreceptor degeneration and irreversible visual loss may occur [4,5].

Substantial advances in vitreoretinal surgery, including pars plana vitrectomy (PPV), scleral buckling, and pneumatic retinopexy, have significantly improved anatomical reattachment rates over recent decades [1,3]. Nevertheless, successful anatomical repair does not necessarily translate into satisfactory functional recovery. Many patients continue to experience reduced visual acuity, metamorphopsia, impaired contrast sensitivity, and other visual disturbances despite complete retinal reattachment [1,3,6]. This discrepancy between anatomical and functional outcomes has generated considerable interest in identifying reliable prognostic biomarkers capable of predicting postoperative visual recovery [2,3].

Optical coherence tomography (OCT) has become an indispensable non-invasive imaging modality in modern retinal practice [2,3]. By providing high-resolution cross-sectional visualization of retinal microarchitecture, OCT enables detailed assessment of individual retinal layers and detection of subtle structural abnormalities that may not be apparent on routine clinical examination [3]. Consequently, OCT has emerged as an important tool for evaluating retinal damage before and after RRD repair and for identifying biomarkers associated with visual prognosis [2,3].

Among OCT-derived biomarkers, the integrity of the ellipsoid zone (EZ), external limiting membrane (ELM), and outer nuclear layer (ONL) has been consistently associated with postoperative visual outcomes [1,2,6,7]. These structures reflect photoreceptor viability and function, and their preservation is generally associated with improved visual recovery following retinal reattachment surgery [1,2,6,7]. In addition, several secondary OCT biomarkers, including persistent subretinal fluid (SRF), intraretinal cystic changes (ICC), hyperreflective foci (HRF), retinal folds, and alterations in retinal thickness, have been investigated as potential indicators of retinal remodeling, inflammation, and postoperative recovery [3,8,9,10,11,12,13].

Despite the growing number of studies evaluating OCT biomarkers in RRD, considerable heterogeneity exists regarding imaging protocols, study populations, timing of OCT assessment, and definitions of individual biomarkers, as emphasized in recent systematic reviews and contemporary evidence syntheses [2,3]. As a result, the relative prognostic value and clinical applicability of many OCT findings remain incompletely defined. Furthermore, ongoing advances in OCT technology and image analysis have improved the assessment of retinal microstructure and expanded interest in OCT-based prognostic modeling and personalized postoperative management [3,14].

The aim of this narrative review is to summarize and critically evaluate the available evidence regarding primary and secondary OCT biomarkers in patients undergoing surgical repair of rhegmatogenous retinal detachment, with particular emphasis on their prognostic significance, clinical applicability, and potential future role in individualized patient management.

## 2. Method

This narrative review was conducted to summarize and critically evaluate the current evidence regarding optical coherence tomography (OCT) biomarkers associated with visual outcomes following rhegmatogenous retinal detachment (RRD) repair. A narrative review approach was selected because the available literature includes heterogeneous study designs, imaging protocols, patient populations, surgical techniques, and outcome measures, which limit the feasibility of a formal systematic review and quantitative synthesis.

A comprehensive literature search was performed using PubMed and Google Scholar databases for studies published up to January 2026. These databases were selected because they provide broad coverage of biomedical and ophthalmic literature and are commonly used sources for evidence synthesis in clinical research. The search strategy included combinations of the following Medical Subject Headings (MeSH) terms and keywords: “rhegmatogenous retinal detachment”, “retinal detachment”, “optical coherence tomography”, “OCT”, “biomarkers”, “ellipsoid zone”, “external limiting membrane”, “hyperreflective foci”, “intraretinal cystic changes”, “persistent subretinal fluid”, “epiretinal membrane”, “predictive factors”, “prognostic factors”, and “visual outcome”.

Eligible studies investigated OCT-derived biomarkers in patients with RRD and evaluated their relationship with anatomical and/or functional postoperative outcomes. Both prospective and retrospective clinical studies were considered. Systematic reviews and meta-analyses were also included to provide contemporary evidence synthesis and to identify additional relevant primary studies not detected during the initial search.

Exclusion criteria included case reports, small case series, experimental animal studies, articles not published in English, and studies lacking a clear association between OCT findings and clinical outcomes. Study selection was independently performed by two authors. Any disagreements regarding study eligibility or relevance were resolved through discussion and consensus among all authors.

Reference lists of all included studies were manually reviewed to identify additional relevant publications. Priority was given to recent studies, particularly those published after 2022, as well as systematic reviews and meta-analyses. Landmark publications were retained when considered essential for understanding the biological basis and historical development of specific OCT biomarkers in rhegmatogenous retinal detachment.

## 3. Results

The principal OCT biomarkers identified in the reviewed studies are summarized in Table 1.

Table 1 was compiled based on evidence from references [1,2,3,4,5,6,7,8,9,10,11,12,13,14,15], including recent advances in artificial intelligence-assisted OCT biomarker analysis in rhegmatogenous retinal detachment [14].

### 3.1. Photoreceptor Integrity (Primary Biomarkers)

Photoreceptor integrity assessed by OCT, particularly the continuity of the ellipsoid zone (EZ) and external limiting membrane (ELM), is widely regarded as one of the most reliable predictors of postoperative visual outcomes in rhegmatogenous retinal detachment [1,2,6,7]. The EZ corresponds to the mitochondria-rich portion of the photoreceptor inner segments, and its structural integrity directly reflects photoreceptor metabolic function. Disruption of the EZ has been consistently associated with poorer postoperative best-corrected visual acuity (BCVA), as demonstrated in both landmark and contemporary studies [1,2,6,7].

More recent evidence has reinforced these findings. Systematic reviews and meta-analyses have confirmed that OCT-derived biomarkers, including EZ integrity and ELM preservation, are strongly associated with postoperative visual outcomes in macula-off RRD, highlighting their continued clinical relevance in modern vitreoretinal practice [2].

The integrity of the ELM represents another critical structural biomarker for visual function following RRD repair. Disruption of the ELM on OCT has been consistently associated with poorer postoperative BCVA [1,2,6,7]. The ELM, formed by junctional complexes between Müller cells and photoreceptors, plays a key role in maintaining photoreceptor alignment and structural stability [15]. It has been suggested that anatomical preservation of the ELM is a prerequisite for subsequent photoreceptor restoration and recovery of the EZ [1,6].

In addition to qualitative assessments, recent studies have focused on quantitative and computational evaluation of photoreceptor integrity. Advances in OCT image analysis and artificial intelligence-assisted assessment have enabled more precise characterization of EZ disruption and other microstructural biomarkers, potentially improving prognostic accuracy in patients undergoing retinal detachment repair [14].

The duration of macular detachment remains a critical determinant of photoreceptor damage. Prolonged separation of the neurosensory retina from the underlying retinal pigment epithelium may result in progressive photoreceptor degeneration, visualized on OCT as persistent disruption of the EZ and ELM [4,5]. This relationship emphasizes the importance of timely surgical intervention to preserve photoreceptor microarchitecture and maximize functional recovery.

Despite some variability among studies related to imaging protocols, timing of OCT acquisition, and patient characteristics, both historical and contemporary evidence consistently supports the role of EZ and ELM integrity as the principal OCT biomarkers for predicting visual outcomes following RRD repair [1,2,6,7].

### 3.2. Secondary OCT Biomarkers

In addition to primary outer retinal biomarkers, several secondary features identified on OCT have been investigated for their potential prognostic value in rhegmatogenous retinal detachment. These include hyperreflective foci (HRF), intraretinal cystic changes (ICC), retinal folds or corrugations, persistent subretinal fluid (SRF), and preretinal hyperreflective dots [3,8,9,10,11,12,13]. Although generally less predictive than outer retinal biomarkers, these findings provide important complementary information regarding retinal remodeling, inflammatory activity, and postoperative recovery.

Hyperreflective foci are small, discrete reflective lesions observed within various retinal layers on OCT. They have been proposed to represent inflammatory cells, activated microglia, lipid-laden macrophages, or photoreceptor debris [8]. Their presence has been associated with retinal inflammation and tissue disruption, and several studies suggest that increased numbers of HRF may be associated with less favorable visual outcomes [9,10]. However, their prognostic significance remains less consistent than that of primary outer retinal biomarkers.

Intraretinal cystic changes are another commonly observed OCT finding, particularly in cases of severe or long-standing retinal detachment. These cystic spaces are believed to reflect chronic retinal edema or degenerative changes affecting the retinal layers [3]. The presence of intraretinal cysts before surgery has been associated with poorer postoperative visual recovery, likely reflecting prolonged retinal dysfunction and structural damage [3]. Persistent cystic changes following surgery may also indicate incomplete macular recovery.

Retinal folds and corrugations represent mechanical distortion of retinal architecture and may be observed before or after retinal reattachment surgery. While mild folds frequently resolve over time, persistent folds involving the macular region may result in metamorphopsia, visual distortion, and reduced visual quality despite successful anatomical reattachment [11,12]. These alterations may interfere with normal photoreceptor alignment and contribute to suboptimal functional outcomes.

Persistent subretinal fluid is another OCT feature frequently observed after successful retinal reattachment. Although it often resolves spontaneously during follow-up, prolonged persistence of subretinal fluid may delay visual recovery and complicate interpretation of postoperative outcomes [3]. The exact relationship between persistent subretinal fluid and long-term functional outcomes remains incompletely understood.

More recently, preretinal hyperreflective dots have been described as a novel OCT finding associated with postoperative retinal remodeling. Emerging evidence suggests that these deposits may represent early proliferative activity on the retinal surface and may be associated with subsequent epiretinal membrane formation [13]. Recognition of these findings may therefore help identify patients at increased risk for postoperative complications and support closer clinical monitoring.

Taken together, secondary OCT biomarkers provide valuable complementary information regarding retinal stress, inflammation, structural remodeling, and postoperative recovery. Although their prognostic value appears less consistent than that of EZ and ELM integrity, they contribute to a more comprehensive assessment of retinal status following rhegmatogenous retinal detachment repair [3,8,9,10,11,12,13].

## 4. Discussion

Optical coherence tomography has fundamentally changed the evaluation of patients undergoing surgical repair of rhegmatogenous retinal detachment by enabling detailed assessment of retinal microstructure and providing valuable prognostic information beyond conventional clinical examination [2,3]. The findings summarized in this review indicate that OCT-derived biomarkers, particularly those reflecting outer retinal integrity, play a central role in predicting postoperative visual outcomes.

Among all investigated biomarkers, the integrity of the ellipsoid zone (EZ) and external limiting membrane (ELM) consistently demonstrated the strongest association with postoperative visual recovery [1,2,6,7]. These findings are biologically plausible because both structures are directly related to photoreceptor viability and function. Preservation of the ELM and EZ reflects maintenance of photoreceptor architecture, whereas disruption of these layers is associated with photoreceptor degeneration and reduced visual potential [1,6,15]. The biological role of Müller cells in maintaining retinal structural integrity and supporting the external limiting membrane has been extensively described in the seminal review by Reichenbach and Bringmann published in 2013. Although this reference predates several recent studies, its fundamental concepts remain widely accepted and have not been substantially revised by subsequent research [15]. Consequently, assessment of these biomarkers has become an important component of postoperative OCT evaluation in patients with RRD.

The present review is consistent with previous studies and recent evidence syntheses that have identified outer retinal integrity as the most reliable predictor of visual outcomes following retinal reattachment surgery [2,3]. While several secondary biomarkers have demonstrated prognostic value, their predictive performance appears less consistent across studies. This variability likely reflects differences in patient populations, imaging protocols, surgical techniques, and timing of postoperative OCT acquisition [3].

Secondary OCT biomarkers nevertheless provide clinically relevant information regarding retinal remodeling and postoperative recovery. Hyperreflective foci may reflect inflammatory activity and retinal stress [8,9,10], while intraretinal cystic changes are often associated with chronic retinal injury and prolonged detachment duration [3]. Similarly, retinal folds and corrugations may contribute to persistent metamorphopsia and reduced visual quality despite successful anatomical reattachment [11,12]. More recently, preretinal hyperreflective dots have emerged as a potentially useful indicator of subsequent epiretinal membrane formation, highlighting the expanding role of OCT in identifying patients at risk for postoperative complications [13].

Recent publications have increasingly emphasized the prognostic role of OCT biomarkers in rhegmatogenous retinal detachment. In particular, the review by Zaidi and Sridhar [3] and the systematic review and meta-analysis by Carlà et al. [2] confirmed the importance of outer retinal integrity, especially EZ and ELM preservation, as key determinants of postoperative visual outcomes. The findings of the present review are consistent with these contemporary analyses while additionally highlighting emerging biomarkers such as preretinal hyperreflective dots and artificial intelligence-assisted OCT assessment [13,14].

Compared with recent reviews and evidence syntheses published in the field, the present review provides an updated overview that incorporates both established and emerging OCT biomarkers associated with visual outcomes after RRD repair [2,3]. In addition to confirming the prognostic importance of EZ and ELM integrity, this review highlights newer OCT features that may improve postoperative risk stratification and patient counseling. The increasing availability of high-resolution imaging technologies and advanced image analysis methods, including artificial intelligence-assisted OCT assessment, is expected to further refine the prognostic utility of OCT biomarkers in clinical practice [14].

Despite significant progress in the field, several challenges remain. Variability in biomarker definitions, differences in image acquisition protocols, and inconsistent timing of postoperative assessments continue to limit direct comparisons among studies [2,3]. Furthermore, many available investigations are retrospective and involve relatively small patient cohorts. These factors emphasize the need for standardized imaging protocols and larger prospective multicenter studies.

Overall, the available evidence supports the routine use of OCT as an essential tool for postoperative evaluation following RRD repair. Assessment of both primary and secondary biomarkers can improve prognostic accuracy, facilitate individualized patient counseling, support clinical decision-making, and enhance understanding of the mechanisms underlying visual recovery after retinal reattachment surgery [2,3].

### 4.1. Clinical Implications

The identification of reliable OCT biomarkers has important implications for the clinical management of patients undergoing surgical repair of rhegmatogenous retinal detachment. Assessment of EZ and ELM integrity may improve the accuracy of postoperative visual prognostication and facilitate more individualized patient counseling [1,2,6,7]. Patients demonstrating preserved outer retinal architecture on OCT generally exhibit greater potential for visual recovery, whereas extensive disruption of these structures may indicate a less favorable prognosis [1,2,6].

OCT biomarkers may also assist clinicians in determining appropriate follow-up strategies. The presence of secondary biomarkers such as persistent subretinal fluid, intraretinal cystic changes, hyperreflective foci, retinal folds, or preretinal hyperreflective dots may identify patients requiring closer postoperative monitoring [3,8,9,10,11,12,13]. Furthermore, recognition of these findings may contribute to earlier detection of postoperative complications, including epiretinal membrane formation and persistent macular dysfunction [3,13].

In addition to qualitative assessment, advances in quantitative OCT analysis and artificial intelligence-assisted image interpretation may further improve prognostic accuracy and risk stratification in patients with RRD [14]. Such technologies may facilitate automated detection of retinal biomarkers and support more personalized postoperative management strategies.

From a practical perspective, incorporation of OCT biomarkers into routine clinical assessment may improve communication between physicians and patients by providing objective information regarding expected visual recovery. This may help establish realistic expectations, optimize postoperative follow-up, and support clinical decision-making throughout the course of treatment [2,3].

As OCT technology continues to evolve, integration of structural biomarkers with emerging computational approaches may further enhance individualized patient care and improve prediction of functional outcomes following rhegmatogenous retinal detachment repair [3,14].

### 4.2. Limitations of Current Evidence

Several limitations should be considered when interpreting the current evidence regarding OCT biomarkers in rhegmatogenous retinal detachment. First, substantial heterogeneity exists among published studies with respect to patient selection, duration of retinal detachment, macular status, surgical techniques, OCT acquisition protocols, and outcome measures [2,3]. This variability complicates direct comparison of results and limits the generalizability of findings.

Second, many available studies are retrospective in design and involve relatively small sample sizes [2,3]. Such methodological limitations may introduce selection bias, reduce statistical power, and increase the risk of confounding factors influencing reported outcomes. Consequently, the strength of evidence supporting some OCT biomarkers remains limited.

Third, the timing of postoperative OCT assessment differs considerably among studies [1,2,6,7]. Since retinal microstructure continues to recover for months after successful retinal reattachment, differences in follow-up duration may significantly influence the interpretation of biomarker restoration and functional outcomes.

Another important limitation is the lack of universally accepted definitions and grading systems for several OCT biomarkers. Variations in biomarker classification, image acquisition settings, and measurement methodology may contribute to inconsistencies among studies and reduce reproducibility across different clinical centers [3].

Furthermore, although emerging biomarkers such as preretinal hyperreflective dots appear promising, the available evidence remains limited and is derived from a relatively small number of investigations [13]. Additional prospective studies are required to validate their prognostic significance and determine their role in routine clinical practice.

Finally, advances in artificial intelligence-assisted OCT analysis have shown encouraging preliminary results; however, these technologies remain insufficiently validated for widespread clinical implementation [14]. Further research is necessary to establish standardized computational approaches and evaluate their performance in large multicenter cohorts.

An additional limitation is the potential for publication bias, whereby studies reporting significant associations between OCT biomarkers and visual outcomes may be more likely to be published than studies with negative or neutral findings, a phenomenon that has long been recognized in biomedical research [16]. Furthermore, most available studies originate from tertiary referral centers and selected patient populations, which may limit the external validity and generalizability of the findings to broader healthcare settings and more diverse patient groups [17].

Future prospective multicenter studies employing standardized imaging protocols, uniform biomarker definitions, and consistent outcome measures are necessary to establish the clinical utility of OCT biomarkers and improve comparability across studies [2,3].

## 5. Conclusions

Optical coherence tomography has become an essential tool for the evaluation and postoperative management of patients undergoing surgical repair of rhegmatogenous retinal detachment. OCT-derived biomarkers provide valuable insights into retinal microstructure and contribute to improved prognostic assessment, individualized patient counseling, and clinical decision-making.

Among currently available biomarkers, the integrity of the ellipsoid zone and external limiting membrane remains the most reliable indicator of postoperative visual recovery, whereas secondary biomarkers offer complementary information regarding retinal remodeling, inflammatory activity, and postoperative complications.

Despite considerable progress, important challenges remain, including heterogeneity among studies, variability in imaging protocols, and the lack of standardized definitions and assessment criteria for several OCT biomarkers. Future large-scale prospective investigations, together with advances in quantitative imaging and artificial intelligence-assisted analysis, may further improve risk stratification and support more personalized postoperative management strategies.

Overall, OCT biomarkers represent a promising and evolving component of modern retinal practice, with substantial potential to enhance prognostic accuracy and optimize functional outcomes following rhegmatogenous retinal detachment repair.

## Figures and Tables

**Table 1:** Summary of Key OCT Biomarkers in Rhegmatogenous Retinal Detachment.

Biomarker	OCT Appearance/Characteristics	Pathophysiological Correlation	Prognostic Significance	Classification
Ellipsoid Zone (EZ)	Hyperreflective band above the RPE; can be continuous, disrupted, or absent.	Photoreceptor inner segment mitochondrial integrity and metabolic health [1,2,6,7].	Strongest predictor of postoperative BCVA; disruption correlates with poor vision [1,2,6,7].	Primary
External Limiting Membrane (ELM)	Fine hyperreflective line above the EZ; represents junctional complexes.	Müller cell-photoreceptor structural stability and alignment [15].	Critical for photoreceptor regeneration; must be intact for EZ recovery [1,2,6,7,15].	Primary
Hyperreflective Foci (HRF)	Small, discrete, highly reflective dots scattered across retinal layers.	Local neuroinflammation, microglial activation, or cellular debris [8,9,10].	Non-specific marker of severe retinal stress; associated with slower recovery [8,9,10].	Secondary
Intraretinal Cystic Changes (ICC)	Hyporeflective fluid-filled spaces within the retinal layers.	Chronic intracellular/extracellular edema or degenerative changes [3].	Preoperative presence indicates long-standing detachment and limited visual recovery [3].	Secondary
Retinal Folds/Corrugations	Undulating or folded appearance of the outer/inner retinal layers.	Mechanical distortion, retinal slippage, or RPE-photoreceptor dysregulation [11].	Induces metamorphopsia and limits visual acuity due to misaligned photoreceptors [12].	Secondary
Preretinal Hyperreflective Dots	Tiny hyperreflective deposits on the inner retinal surface (ILM).	Early proliferative activity and cellular seeding post-vitrectomy [13].	Highly associated with subsequent epiretinal membrane (ERM) formation [13].	Secondary (Novel)

## Data Availability

No new data were created or analyzed in this study. Data sharing is not applicable to this article.
